# A High Throughput Amenable Arabidopsis-*P. aeruginosa* System Reveals a Rewired Regulatory Module and the Utility to Identify Potent Anti-Infectives

**DOI:** 10.1371/journal.pone.0016381

**Published:** 2011-01-21

**Authors:** Suresh Gopalan, Frederick M. Ausubel

**Affiliations:** 1 Department of Molecular Biology, Massachusetts General Hospital, Boston, Massachusetts, United States of America; 2 Department of Genetics, Harvard Medical School, Boston, Massachusetts, United States of America; Instituto de Biología Molecular y Celular de Plantas, Spain

## Abstract

We previously demonstrated that in a metasystem consisting of Arabidopsis seedlings growing in liquid medium (in 96 well plates) even microbes considered to be innocuous such as laboratory strains of *E. coli* and *B. subtilis* can cause potent damage to the host. We further posited that such environment-induced adaptations are brought about by ‘system status changes’ (rewiring of pre-existing cellular signaling networks and components) of the host and the microbe, and that prolongation of such a situation could lead to the emergence of pathogenic states in real-life. Here, using this infection model, we show that the master regulator GacA of the human opportunistic pathogen *P. aeruginosa* (strain PA14) is dispensable for pathogenesis, as evidenced by three independent read-outs. The gene expression profile of the host after infection with wild type PA14 or the *gacA* mutant are also identical. GacA normally acts upstream of the quorum sensing regulatory circuit (that includes the regulator LasR) that controls a subset of virulence factors. Double mutants in *gacA* and *lasR* behave similar to the *lasR* mutant, as seen by abrogation of a characteristic cell type specific host cell damage caused by PA14 or the *gacA* mutant. This indicates that a previously unrecognized regulatory mechanism is operative under these conditions upstream of LasR. In addition, the detrimental effect of PA14 on Arabidopsis seedlings is resistant to high concentrations of the aminoglycoside antibiotic gentamicin. These data suggest that the Arabidopsis seedling infection system could be used to identify anti-infectives with potentially novel modes of action.

## Introduction

New and re-emerging microbial diseases are a serious threat to human and agricultural health. In depth analysis of indigenous microbes in a host makes it increasingly evident that host-microbe, and microbe-microbe interactions adapted to specific niches play an important role in the health of mammalian hosts [1], [2], [3], [4]. On the other hand, even when a host mounts a response to a pathogen, it is becoming increasingly clear that there is a delicate balance between inflammation and immunity and that there is evolutionary pressure to maintain an appropriate balance between these states [5], [6]. We posited that ‘system status changes’ during the early stages of certain environmentally-induced host-microbe interactions (rewiring of pre-existing cellular signaling networks and components) may underlie the ability of a microbe to establish a new pathogenic interaction with a host, which from the host's perspective can be viewed as an environmentally-induced “maladaptaion”. Continued existence of such states could lead to emergence of new infectious agents [7]. Once established, the microbes can further adapt through lateral gene transfer and other modes of genetic fixation to evolve increased adaptation to a host environment [8]. Certain nosocomial infections, zoonotic infections, hygiene related emergence of infectious agents, and niche alteration induced diseases by indigenous microbes are likely to emerge in this manner.

Since such modes of emergence are difficult to study in their natural settings and in a realistic time frame, we established a model system of *Arabidopsis thaliana* seedlings growing in liquid medium in 96 well plates interacting with a variety of microbes. In this environment a human opportunistic pathogen (*Pseudomonas aeruginosa*), a plant pathogen (*Pseudomonas syringae*), as well as two commonly considered innocuous laboratory microbes, *Escherichia coli* (strain Dh5α) and *Bacillus subtilis,* cause potent and characteristic host damage. Damage by these microbes was demonstrated by alterations of the activity of a constitutively expressed luciferase in the host, as well as by using fluorescent probes indicative of altered host cell permeability. Of particular interest were the facts that (i) *B. subtilis*-induced host damage was very potent, (ii) a mutant of the quorum sensing regulator LasR of *P. aeruginosa* was attenuated in virulence and altered in the characteristic type of cell type specific damage that it elicited in comparison to the wild-type, and (iii) the strong attenuation of *P. syringae*-induced damage by a mutation affecting the type III secretion system. On the host front, cell patterning defects (indicative of alterations in intercellular signals and/or hormonal status altered in this system), as well as yet to be characterized defect(s) affecting execution of immune response pathways were also demonstrated [7].

Here we explore some additional features of *P. aeruginosa* – host interactions in this system. The data presented here reveal a novel regulatory alteration in the quorum sensing module of the microbe. In addition the data reveal extreme resistance of *P.aeruginosa* induced host damage to high concentrations of the antibiotic gentamicin added to this system.

## Results

### The master regulator GacA is dispensable for *P.aeruginosa* induced damage to Arabidopsis seedlings

**Figure 1:**
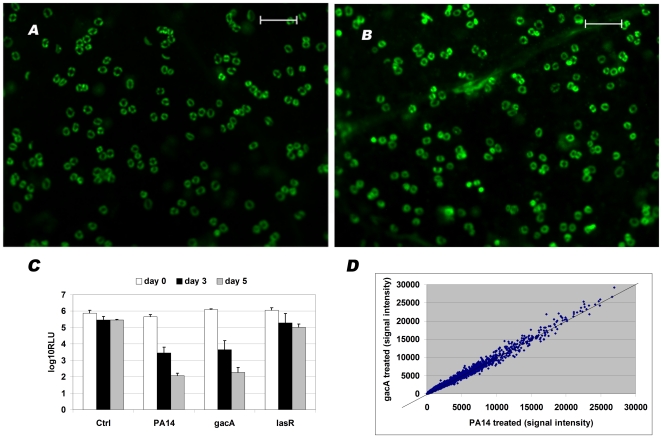
The two-component regulator GacA is dispensable for virulence in this Arabidopsis seedling – *P. aeruginosa* system. **A & B**. Microscopic observation of leaf tissue from seedlings infected with *P. aeruginosa* PA14 (panel **A**) or PA14Δ*gacA* (panel **B**) three days post-infection and stained with Sytox Green® reveals characteristic and similar staining patterns of stomatal guard cells by both strains. Scale bar represents 100 µM. **C**. The luciferase activity (log_10_ of relative luminescence unit) in Arabidopsis seedlings designed to express the enzyme constitutively in untreated seedlings or after infection with PA14, PA14Δ*gacA*, PA14Δ*lasR* at 0, 3 and 5 dpi. **D**. The trancriptome of Arabidopsis seedlings 1 dpi indicate identical response to PA14 and PA14Δ*gacA* (indicated as gacA in the figure). Shown are average values of signal intensity of two replicate experiments for each treatment. The diagonal line across the graph indicates the expected line of no change.

We previously reported that PA14 causes near complete abrogation of a constitutively expressed luciferase reporter gene in Arabidopsis seedlings by five days after infection [7]. This decrease in luciferase activity was reflected in visual symptoms indicative of host death in this host-microbe combination. Of many PA14 mutants tested, only mutation of the quorum sensing regulator LasR showed an attenuation of disease symptoms. In this report we tested the effect of a mutation in the global regulator GacA, which has been shown to act upstream of LasR [9]. The visual symptoms after treatment of seedlings with PA14 and PA14Δ*gacA* were nearly identical (data not shown). When the treated seedlings were stained with Sytox Green® (Invitrogen, CA) to monitor membrane permeability (another aspect of host damage), the microscopic images were identical. As had been shown with PA14 earlier [7] (and Fig. 1A), Sytox Green® staining in PA14Δ*gacA-*infected leaves was characteristically present in most stomatal guard cells and unexpectedly the staining was evenly distributed throughout these cells (Fig. 1B). A similar result (i.e., identical response to PA14 and PA14Δ*gacA* treatment) was observed when abrogation of luminescence, designed to be expressed constitutively in the seedlings, was used as a read-out (Fig. 1C).

Since in both the Sytox Green® and luciferase assays PA14 and the Δ*gacA* mutant behaved very similarly, we checked gene expression patterns in the host. For this we used the ATH1 gene expression chip from Affymetrix (CA). As shown in Fig. 1D (Table S1), the patterns of gene expression one day post infection with either wild type PA14 or the *gacA* mutant from two independently conducted experiments were essentially identical. These data are consistent the the luciferase and Sytox Green® staining experiments described above. The transcriptomes following PA14 or PA14Δ*gacA* infection shown in Fig. 1D were markedly different from untreated seedlings or from treatments with other bacteria (data not shown).

### GacA is dispensable for LasR dependent phenotype in the seedling infection model

**Figure 2:**
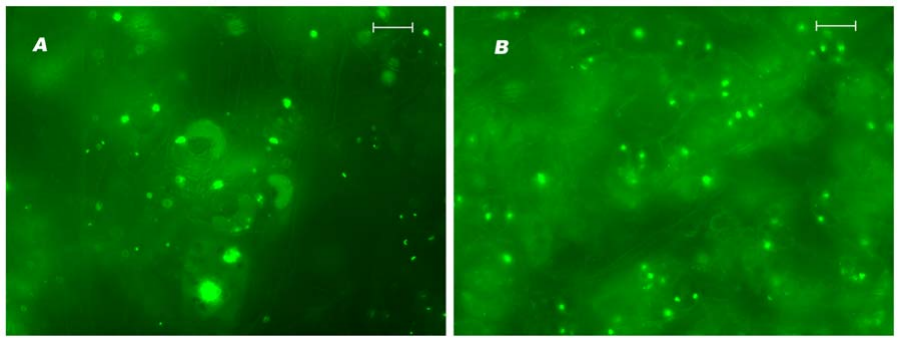
Seedlings treated with *P. aeruginosa* PA14Δ*lasR* and PA14Δ*gacA*Δ*lasR* show identical Sytox Green® staining pattern of leaves. Seedlings were treated with PA14Δ*lasR* (panel **A**) or PA14Δ*gacA*Δ*lasR* (panel **B**) and stained with Sytox Green® three dpi. Shown are the observed staining patterns (diffuse staining pattern on many cell types) of the leaves observed under the microscope. Seedlings treated with PA14 or PA14Δ*gacA* show characteristic intense staining pattern of the stomatal guard cells. Scale bar represents 20 µM.

Because GacA has been shown to act upstream of LasR [9], we constructed a double mutant in *gacA* and *lasR* to carry out epistasis analysis. Two independent double mutants were used to check for the characteristic stomatal guard cell staining pattern after staining with Sytox Green (Invitrogen, CA) – both yielded identical results. As shown in Fig. 2 the PA14Δ*lasR* and PA14Δ*gacA*Δ*lasR* double mutant showed diffuse staining of different cell types in contrast to bright staining specifically in stomatal guard cells in leaf issue from seedlings treated with PA14 or PA14Δ*gacA* (data not shown at this magnification). The simplest explanation of the combination of these two results is that LasR functions independently of GacA in the Arabidopsis seedling – PA14 pathogenesis model.

### Increased antibiotic resistance displayed by *P. aeruginosa* interacting with the host in this system

**Figure 3:**
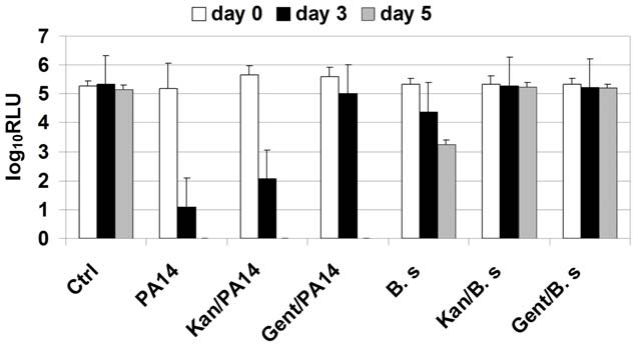
Increased resistance of *P. aeruginosa* PA14 induced host damage to the antibiotic gentamicin. The luciferase activity (log_10_ of relative luminescence unit) in Arabidopsis seedlings designed to express the enzyme constitutively and treated with kanamycin (50 µg/ml) or gentamicin (100 µg/ml) prior to addition of *P. aeruginosa* PA14. The response to *B. subtils* (B. s) treated under similar conditions is also shown.

In addition to acquiring genetic elements from their community that confer new antibiotic resistance, it is well known that microbes adapt in ways that can be considered ‘system status changes’. Thus, we tested the response of this system when *P.aeruginosa* was added in the presence of two antibiotics one at levels lower than the MIC (kanamycin, 50 µg/ml) and other much higher than they are normally sensitive to (gentamicin, 100 µg/ml) in rich medium. Earlier it has been shown that the MIC of PA14 to kanamycin is around 100 µg/ml at 25°C [10]. Further, PA14 is normally completely susceptible to gentamicin at 15 µg/ml. This is evident by the fact that a whole library of transposon insertion in genes of PA14 was constructed by selecting at this concentration [11]. There was no effect of kanamycin under these conditions (i.e., no increased susceptibility). However, as shown in Fig. 3, even at 100 µg/ml, gentamicin only appeared to have a marginal effect on the virulence of PA14 in the Arabidopsis pathogenicity assay. There was a modest time delay in the appearance of visual symptoms (data not shown) and the abrogation of constitutive luciferase expression when treated with gentamicin at 100 µg/ml. There was no evidence for the formation of a PA14 biofilm on the surface of the Arabidopsis leaves based on careful microscopic analysis of *P. aeruginosa* expressing a green fluorescence protein (GFP) in this submerged seedling infection system (data not shown). Under the same conditions as this experiment, the potent damage to the host by *B. subtilis* was completely abrogated by both these antibiotics as seen through visual observation of seedling health (data not shown) and the expression of luciferase activity (Fig. 3). This latter result with *B. subtilis* indicates that the antibiotic is active under these experimental conditions. In addition, *P. aeruginosa* (PA14) is highly susceptible to gentamycin at concentrations less than 10 µg/ml in MS medium alone or in conditioned medium, i.e., after growth of seedlings (data not shown).

## Discussion

We posited that early stages of emergence of certain host-microbe maladaptations involve environment induced alterations of host and microbial processes, typically characteristic of the kind of adaptation. A metasystem of framework model organisms established earlier serves as a good model to study fundamental principles of the early stages of such emergence of adaptations that leads to new infectious diseases [7]. This system consisting of Arabidopsis seedlings interacting with a number of microbes, revealed conditions where even common laboratory microbes such as *E. coli* and *B. subtilis* caused potent host damage.

The 96-well format, and multiple readouts of host damage and metabolic alterations (through luminescence, chlorophyll fluorescence and Sytox Green® fluorescence indicative of membrane damage, all of which are quantitative measures) makes this an excellent system amenable to a wide variety of automation assisted high-throughput screens. In addition, this model should aid in building a fundamental knowledge reference framework using system wide measurements and integrating currently available as well as emerging extensive knowledge base for the constituent organisms of this system.

**Figure 4:**
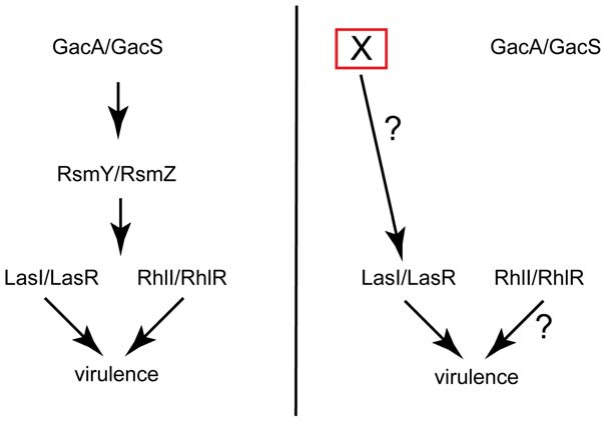
An alternative regulatory mode circumventing the role of GacA upstream of the quorum sensing regulator LasR. The left panel indicates the canonical model for the relationship in *P. aeruginosa* of GacA and its role upstream of the quorum sensing regulator LasR (that affects a subset of virulence effectors). The panel on the right is a depiction of the yet to be understood alternative regulatory circuit operative in this host-pathogen system. A proposed unknown effector substituting for the role of the dispensable GacA is indicated as a component X, upstream of LasR.

Here we explored the utility of the system further by using it to reveal a previously unrecognized regulatory mechanism operative upstream of the key quorum sensing regulator LasR in *P. aeruginosa*. This mechanism seems to circumvent the need for GacA, a master regulator that has been shown to act upstream of LasR [9]. The role of GacA in controlling a subset of virulence factors is also known in other microbial pathogens [12], [13]. Indeed, in a *C. elegans* model of host-microbe interaction involving *P. aeruginosa* PA14, the role of GacA is very prominent and the pathogenicity of a PA14Δ*gacA* mutants is significantly abrogated [14]. In a burned mouse model as well as an Arabidopsis leaf infection model PA14Δ*gacA* was significantly affected in virulence [15]. In contrast, in the Arabidopsis seedling- *P. aeruginosa* system used in this study, a GacA independent regulatory circuit acting upstream of LasR was revealed. A PA14Δ*lasR* mutant and two independent PA14Δ*gacA*Δ*lasR* double mutants behaved similarly in the assay used here, as shown by the fact that both of these classes of mutants abolished the characteristic staining of stomatal guard cells after infection using the membrane impermeable dye Sytox Green®. In addition, it should be highlighted that both wild type PA14 and the PA14Δ*gacA* mutant behaved similarly by four different read outs (visual phenotype, expression of constitutively expressed luciferase activity of the host, characteristic cell type specific staining by Sytox Green®, and host transcriptome) post infection. These results imply complete dispensability of a role for GacA for the function of LasR, and suggests compensatory mechanisms operative under these conditions. This result is shown in a pictorial model in Fig. 4. This result also suggests that such environment-induced rearrangement and utilzation of pre-existing modules and components may play a significant role in natural infections. This system thus appears to be capable of highlighting some previously uncharacterized regulatory circuits, in addition to having other desirable experimental features highlighted earlier [7].

Many pathogens, especially well studied nosocomial infections and infections in immune compromised patients seem to rapidly acquire resistance to a number of drugs. This typically happens by two broad routes, lateral acquisition of antibiotic resistance genes and other genetic adaptations over a period of time, and through alteration that can be considered ‘system status changes’. Some forms of previously classifications, including phenotypic antibiotic resistance involving biofilm formation, phase variation and the activation of efflux pumps etc. [10], [16], can be explained mechanistically through ‘systems status changes’. Thus having high-throughput amenable systems suited for large scale identification of host and pathogen components contributing to the latter route, as well as the ability to screen for novel anti-infective compounds with far higher potency should be a significantly advantageous. The capability of *P. aeruginosa* to cause disease at concentrations of the antibiotic gentamicin over 10 fold above the concentration that normally prevents its growth demonstrates the potential to identify effective anti-infectives to prevent the adaptation in this system.

The enhanced resistance of *P. aeruginosa* pathogenesis in this and other systems could result from the ‘system status changes’ of the host and of the microbe, and environment induced alterations of antibiotic effectiveness. Specifically, we have provided evidence that a well studied genetic cascade has been rewired in *P.aeruginosa* under these conditions. Earlier, we had provided several lines of evidence that point toward multiple aspects of host alterations that prevent execution of host immune response [7]. These previous data, together with the high-throughput advantages highlighted above, indicate that different screens (chemical compounds as well as genetic) using the *P.aeruginosa* – Arabidopsis seedling interaction system could reveal strong resistance inducing components and compounds. Such screens have the potential to highlight anti-infectives that act through novel modes of action. Earlier it has been shown using a *C. elegans*-*E.faecalis* system that high throughput chemical screens using host-microbe systems can identify antimicrobials as well as compounds that seem to potentiate the host immune system [17].

In summary, this work (i) reinforces the proposition and concept that environment induced ‘system status changes’ play a major role in adaptation of hosts and microbes and provide an opportunity for the emergence of new infectious diseases, and (ii) establishes this high-throughput system of Arabidopsis seedlings interacting with *P. aeuginosa* as a powerful tool to identify potent anti- infectives that potentially act through novel modes of action.

## Materials and Methods

### Plant and bacterial growth and assays

Plant and bacterial growth and infections and other assays were essentially as described earlier [7]. The seeds used were of genotype Col-0 designed to express the luciferase gene (hRluc) under a constitutive 35S CaMV promoter. The sterilized seeds were grown in 96 well plates at 22°C. On day 11, 10 µl of 0.0002 ABS600 of exponentially growing cultures of appropriate bacteria were added to each well and incubated at 25°C until the indicated time points. The whole experiment was done at a light intensity of 75 µE. For luminescence readings the substrate coelenterazine (0.5 µM) was added to each well and incubated in the dark for 20 mins, prior to reading in the Topcount NXT (Perkin Elmer, CA). For fluorescence experiments Sytox Green® (1 µM) was added to each well and incubated in the dark at room temperature for 3 h to 12 h prior to microscopic observation.

The PA14Δ*gacA* strain used and the *lasR* insertion cassette and mutant construction are as described earlier [15], [18]. The two PA14Δ*gacA*Δ*lasR* mutants used were confirmed for insertions in the *gacA* and *lasR* genes by PCR. When antibiotics were used in the host-pathogen assays, they were added at indicated concentrations 12 hours prior to inoculation with bacteria. In Fig. 1C the TopSeal® (Perkin Elmer, MA) was not removed between the day 3 read and before substrate addition for the day 5 read. This resulted in residual luciferase activity on day 5 when treated with PA14 or PA14Δ*gacA*. A typical result was complete abrogation of seedling luciferase activity by day 5 when treated with these two strains.

### Microarray analysis of transcriptome

The ATH1 GeneChip® microarrays (Affymetrix, CA) were used to study the transcriptome. Two replicates of transcriptome from seedlings treated with PA14 or PA14Δ*gacA* (using RNA extracted from seedlings from two independent experiments) from 1 dpi were generated.

Seedlings grown and treated as above on day 10 with an exponentially growing culture of PA14 or PA14Δ*gacA* were flash frozen in liquid nitrogen 24 h post-infection and used for total RNA extraction.

The microarray data were generated starting from total RNA by the Harvard Medical School Biopolymers Facility, according to protocols provided by the Affymetrix GeneChip analysis technical manual. Arrays were scanned using GeneArray® 3000 7G Scanner. The results were processed by the MAS5® algorithm in GCOS® (Affymetrix, CA), with all arrays scaled to a median signal intensity of 500. For the results shown in the graph, probesets (genes) that were called “Absent” in all four arrays (two treatments, two replicates each), as well as those that had a signal value less than 50 in all four replicates were filtered out from data shown in the graph in Fig. 1D. The MAS5® processed raw data before and after filtering are shown in Table S1. As mentioned in the “Results” section, the expression profile of the results presented here are significantly different from untreated seedlings or from seedlings treated with other bacteria (data not shown). The microarray data presented in this publication have been deposited in the MIAME compliant data base – NCBI's Gene Expression Omnibus (GEO, http://www.ncbi.nlm.nih.gov/geo/), and are accessible through GEO Series accession number GEO26084.

## Supporting Information

Table S1 **Transcriptome of host (Arabidopsis seedlings) 24 h after treatment with** ***P. aeruginosa*** **(strain PA14) wild type or a** ***gacA*** **mutant.** Shown are values of expression of transcripts from two replicates determined using ATH1® chips (Affymetrix, CA) before and after filtering low values as described in ‘Materials and Methods’ section.
(XLS)
